# Supplementary material for: Autotaxin Has a Negative Role in Systemic Inflammation
Source: Int J Mol Sci. 2022 Jul 18;23(14):7920. doi: 10.3390/ijms23147920 (PMC9322786; doi:10.3390/ijms23147920)
Supplement: Supplementary file 1 [file ijms-23-07920-s001.zip › ijms-1766246-supplementary.pdf]

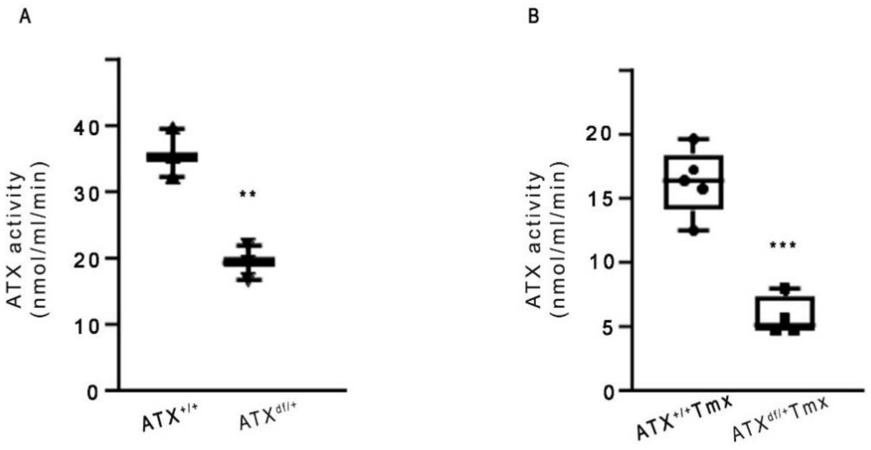

Figure S1. Genetic deletion of *Enpp2* (ATX) in mice decreases serum ATX levels. Decreased ATX activity levels were detected in heterozygous ATX knockout mice (A) and in mice bearing inducible inactivation of ATX (B), compared to their littermate controls. ATX activity was measured with the TOOS assay. Comparisons were assessed using an unpaired t-test, after conducting a Shapiro-Wilk normality test. ** signifies $p<0.01$, *** $p<0.001$.

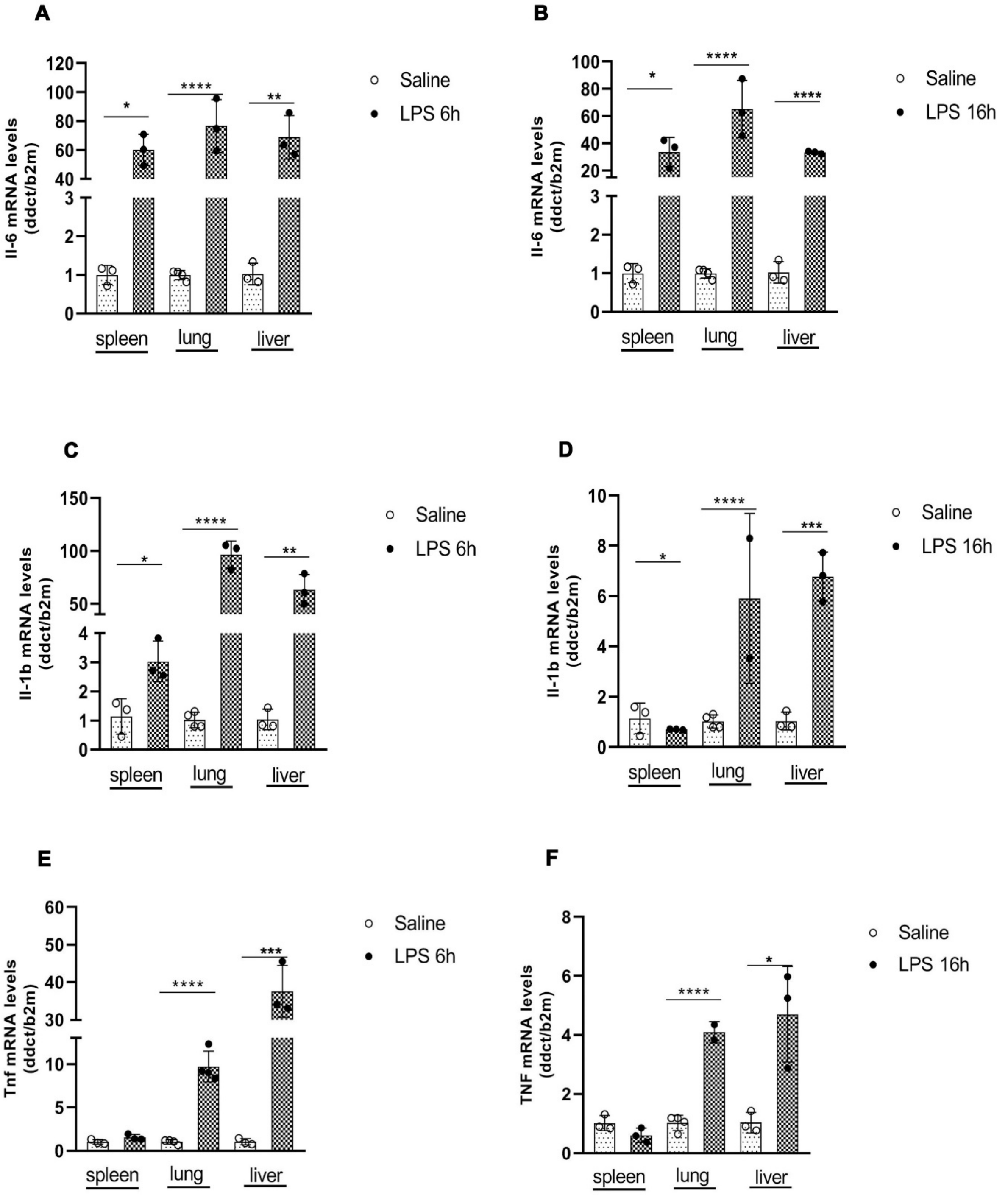

Figure S2. Systemic LPS administration stimulates pro-inflammatory gene expression in different tissues of mice. IL-6 (A, B), IL-1b (C, D) and TNF (E, F) mRNA levels in the indicated mouse tissues, 6 and 16 hours post LPS administration were quantified with Q-RT-PCR. Values were normalised to the corresponding Beta2-microglobulin (b2m) ones (ΔΔct). Statistical significance was assessed with t-test; */**/***/**** denote a p-value of $<0.05/0.01/0.001/0.0001$ respectively.
